# Residential Evictions by Life Course, Type, and Timing, and Associations with Self-rated Health: Social Epidemiology to Combat Unjust Residential Evictions (SECURE) Study

**DOI:** 10.1007/s11524-025-00977-w

**Published:** 2025-05-14

**Authors:** Shawnita Sealy-Jefferson, Loretta J. Ross, Tamika Anderson-Mays, Kyra Sanders, Roquesha Oneal, JoAnn M. Booth, Jacqueline Brown, Swati Mishra, Tiffany N. Ford, Kierra Barnett, Shibani Chettri, Chinenye Bosah, Mindy Hoang, Scarlett Bellamy

**Affiliations:** 1 Ohio State University, 1841 Neil Avenue, Columbus, OH 43210 USA; 2 Smith College, Northampton, USA; 3 Social Epidemiology to Combat Unjust Residential Evictions (SECURE) Study Community Advisory Board, Detroit, USA; 4 University of Illinois Chicago, Chicago, USA; 5 University of Maryland, College Park, USA; 6 Nationwide Children’s Hospital, Columbus, USA; 7 University of Cincinnati, Cincinnati, USA; 8 Wayne State University, Detroit, USA; 9 Drexel University, Philadelphia, USA; 10 Boston University, Boston, USA

**Keywords:** Court-ordered evictions, Illegal evictions, Evictions during childhood, Black women, Descriptive epidemiology, Social epidemiology, Self-rated health, Relative self-rated health, Structural racism, Trauma

## Abstract

Few existing data sources quantify the magnitude of court-ordered and illegal residential evictions, among historically marginalized groups. We describe the Social Epidemiology to Combat Unjust Residential Evictions (SECURE) Study (2021–2024; *n* = 1,428; 91.1% response rate) methodology and participant characteristics. Univariable and multivariable statistics including Spearman correlations were used to describe data. Unadjusted and adjusted modified Poisson regression with robust error variance estimated relative risk (RR) and associated 95% confidence intervals (95% CI) for associations between five eviction measures and self-rated health (SRH), and self-rated health relative to most similarly aged peers (RSRH). A quarter of the participants reported experiencing an eviction as a child (*n* = 354). Over half of the study sample reported ever experiencing a court-ordered (*n* = 432) and/or an illegal eviction (*n* = 360). In the past 2 years, 15.2% of the sample reported experiencing a court-ordered (*n* = 122) and/or illegal eviction (*n* = 95). Eviction during childhood, and ever experiencing both court-ordered and/or illegal eviction was associated with between 12 and 17% higher risk of poor SRH, and childhood eviction and ever experiencing illegal eviction was associated with between 34 and 37% higher risk of worse RSRH among reproductive age Black women. More community-partnered research using participatory action research methods are needed to understand and intervene upon the health impacts of residential evictions among disproportionately impacted groups.

## Introduction

While limited empirical evidence exists to understand individual experiences of court-ordered and illegal eviction and their impact on population health inequities [1], the research shows that Black women with children are unjustly impacted [2]. Calls have been made for more rigorous descriptive epidemiologic studies, of both exposures and outcomes, to characterize the distributions of health and wellbeing, as well as harmful, and protective exposures among marginalized populations [3–5]. Further, while epidemiologic evidence on predictors of self-rated health is well documented, few existing studies focus on residential eviction by type, life-course, and timing, or use within group analyses among the most at-risk group. Therefore, we present the methodology and descriptive analyses for the Social Epidemiology to Combat Unjust Residential Evictions (SECURE) study, as well as associations between five eviction experiences and two measures of self-rated health. The goal of SECURE study was to document the magnitude and intensity of court ordered and illegal residential evictions as well as their impact on Black women, families, and communities in Metro-Detroit, Michigan (including Wayne, Oakland, and Macomb counties). Specifically, using an online survey, we collected data on: individual experiences of court-ordered and illegal evictions, mental and physical health, social, behavioral, and psychosocial factors, childhood experiences, clinical, and other factors.

## Methods

### Intention for the Work

We began the work with humility and a deep respect for the humanity and dignity of Black women. We intended to listen, give grace to, trust, and value the contributions to knowledge generation of the target population.

### Theoretical Foundation

SECURE study was grounded in and informed by several theoretical frameworks, including reproductive justice [6], intersectionality [7], ecosocial theory of disease distribution [8, 9], critical race theory [10, 11], Black feminist thought [12], and epistemic exclusion theory [13, 14]. The theoretical frameworks helped us make decisions about what to include in our work and how to include it, as well as to align our behaviors and values in conducting the research.

### Community Advisory Board

The SECURE Study Community Advisory Board (CAB) is a multigenerational group and is comprised of Black women who are international and local leaders including those whose work focuses on housing and reproductive justice, advocacy, education, health care, property management, journalism, as well as those with lived experience of eviction. CAB members were identified and invited by the principal investigator (PI) and were involved in study planning and decision-making, recruitment, community engagement, interpretation of study findings, and dissemination of results (as named coauthors). The CAB meetings with the PI were monthly and/or as needed. We used a project management software to communicate between meetings and to coordinate our work together. We used a wide range of participant recruitment strategies including (but not limited to) social media, direct community engagement by the PI and CAB, word of mouth, billboards, and printed fliers.

### Setting

#### Participant Eligibility

SECURE study eligibility was not contingent on the outcomes or exposures of interest. Eligibility for SECURE study was limited to those who (1) self-identified as African American/Black woman; (2) were current residents of Wayne, Oakland, or Macomb County, Michigan; (3) between 18 and 45 years old; and (4) reported being a parent or having a parenting role.

#### Online Survey Verification Methods

An online screening survey administered through Qualtrics was used to determine presumptive eligibility, and was deployed from July 18, 2021 to July 31, 2024. Qualtrics security configurations were used to screen out bots, duplicate submissions, and fraudulent responses. Qualtrics logic based geoIP using latitude and longitude coordinates were used to filter out individuals not physically in Metro-Detroit, Michigan, USA. We had a required verification step as part of the original research protocol that included a brief zoom meeting with the PI or research staff to confirm participant identity and eligibility. Our eligibility verification step was fortunate, given what we experienced as *extreme* fraudulent attempts at participation (*n* = 7002 online screening survey results from bots and people who were located outside of the Metro-Detroit, MI eligibility area, in states across the USA, and *multiple* other countries). Only *n* = 30 people scheduled and participated in a verification meeting who were determined to be ineligible. After eligibility was confirmed, participants were allowed to ask questions, gave online informed consent, and completed a telephone or self-administered online survey. Before and/or during verification meetings, the PI cross-checked for duplicate names, emails, and/or phone numbers in screening surveys, the completed verification meeting logs, consent forms, completed surveys, and incentive records to ensure that each person participated only once.

#### Online Survey

The survey was designed to take 45–60 min to complete, and participants were given 2 weeks to complete the survey that was emailed to them. If a participant requested a telephone interview to complete the survey, an appointment was scheduled at the convenience of the participant. Most telephone interviews lasted between 1.5 and 2 h. After surveys were completed, participants received an electronic gift card for $50. The final survey sample size was *n* = 1428 participants, which represented 91.1% of those who were confirmed to be eligible (*n* = 1567). The a priori planned sample size was *n* = 1600 participants. SECURE study was approved by the Ohio State University Office of Responsible Research Practices.

#### Exposures

The legal forced removal of a tenant from a rental property with a court-order is defined as a court-ordered eviction. Eviction law varies by jurisdiction [15], and for the geographic context in which our study takes place, an illegal eviction refers to the forced residential displacement of a tenant from a rental property by the owner or their representative, without a court order [16]. Informed by and expanding the existing literature on eviction [17], we used community based participatory action research methods [18] to identify five pertinent eviction measures, by type (court-ordered and illegal) life course (childhood and adulthood) and timing (ever and in the past 2 years). Participants answered (yes/no) to the following questions: (1) “when you were growing up (before your 18th birthday), did you and your family ever experience an eviction (being put out either after a court-order or not?)”, (2) “Have you ever experienced a court-ordered eviction (your landlord took you to court and the judge ordered you to move)?”, (3) “Have you ever experienced an illegal eviction (when your landlord did not take you to court, but still evicted you/put you out)?”, (4) “In the past two years, have you had a court-ordered eviction?”, and (5) “In the past two years, have you been evicted, but the landlord did not take you to court?”.

#### Outcomes

Self-rated physical health (SRH) [19] and an age-comparative relative self-rated health (RSRH) measure (compared to others of the same age) [20] were both examined given the dearth of research on their associations with multiple measures of eviction in our target population. For SRH, participants were asked “In general, how would you rate your overall physical health at this time?” with the following response categories: poor, fair, good, very good, and excellent. For RSRH, participants answered the question: “How would you rate your physical health compared to most people your age?”, with responses: a lot better, a little better, average, a little worse, or a lot worse.

#### Covariates

Informed by existing literature [21–23] and to avoid over-adjustment bias [24], we considered the following self-reported variables as covariates: age (years), annual household income (17 categories, with $4999 increments from < $5000 to $59,999, and $9999 increments from $60,000 to $100,000 or more), employment status (employed or unemployed), housing tenure (owned by you or someone in this household with a mortgage or loan, owned by you or someone in this household free and clear (without a mortgage or loan), occupied without payment of rent, rented, other (open-ended question, and including more than one of the previously listed categories), and number of live born children. Table 1 provides details about the other descriptive variables included in this analysis.

**Table 1:** Social Epidemiology to Combat Unjust Residential Evictions (SECURE) study descriptive variables and measurement

Variable	Measurement
Relationship status	• Married, cohabiting, divorced, separated, widowed, never married
Educational attainment	• 9 categories from 1 to 8th grade through doctorate degree
Financial situation today	• How would you describe your family’s financial situation today? • 5-point Likert scale from very poor, not enough to get by to well to do
Sexual orientation	• 8 categories (asexual, bisexual, gay, heterosexual, lesbian, pansexual, queer, questioning or unsure)
Mental and emotional health	• 5-point Likert scale from excellent to poor
Sleep quality	• 4-point Likert from very good to very bad
Religiosity	• 4-point Likert from very religious to not at all religious
Skin tone	• 7 categories from very light to very deep
Overall level of trust	• “Generally speaking, would you say that most people can be trusted or that you can’t be too careful in dealing with people?”
Expanded adverse childhood experiences (ACEs) scale [34]	• “Think about experiences that you had growing up (before your 18th birthday). Please tell us whether you had any of these experiences.” • The original 10 ACES, plus witnessing violence, experiencing racism, neighborhood danger and distrust, bullying, and foster care experience • Yes/no
Emotional support, social network support, volunteer activities in the past year, provision of financial and non-financial support to others, self and partner incarceration, current neighborhood gentrification, and perceived benefits and disadvantages of gentrification	• Yes/no
Housing discrimination [26]	• “Have you ever been discriminated against (treated unfairly) when you were trying to buy or rent a house or apartment?” • Yes/no

#### Statistical Methods

Cut points of descriptive variables were based on sample distributions. Age was categorized as 18–19, 20–24, 25–29, 30–34, and 35 + years. Annual income was dichotomized at the median (< $35,000, ≥ $35,000/year). SRH was dichotomized as good (including excellent, very good, and good) versus poor (including fair and poor). RSRH was dichotomized as better (including a lot better, a little better, and average) and worse (a little worse, or a lot worse). Housing tenure was modeled as a five-category variable representing the original reported categories, number of live born children was categorized as 0 or 1 + , and employment status was dichotomous (employed/unemployed). Univariable and multivariable statistics, including Wilcoxon rank sum and chi square tests were used to detect differences in continuous and categorical variables. Spearman correlations estimated relationships between five eviction variables. We present descriptive results in the total sample and stratified by our five eviction measures, to support future hypothesis generation. Given non-rare outcome measures, we used modified Poisson regression analyses with robust error variance to estimate relative risk (and associated 95% confidence intervals) of poor SRH and worse RSRH, associated with our five eviction variables in unadjusted and adjusted models. In models for both court-ordered and illegal eviction (ever and in the past 2 years), we excluded participants who reported experiencing an eviction before age 18 (*n* = 354), to reduce the impact of people who were trapped in a lifelong cycle of eviction beginning in childhood, consistent with existing literature [21]. Missing data ranged from 0 to 6.6% for all study variables (Table 1), and we used listwise deletion. SAS version 9.4 for Windows was used for statistical analysis (SAS Institute Inc., Cary, NC, USA).

## Results

### Descriptive Statistics in the Total Sample

A total of 1428 women were included in the study, and the mean age of participants was 35 years (standard deviation (SD): 6.45, range 18–46). Half of the participants reported never being married (*n* = 715), and 19% reported being currently married (*n* = 273) (Table 2). Most participants (88%) reported having a prior live birth (*n* = 1261), and the mean number of live births was 2.3 (standard deviation: 1.63, range 0–12). More than 70% of participants had more than 12 years of education (*n* = 1015), with the highest proportion of participants attending some college (34.7%). The median annual income category reported in the sample was $30,000–$34,999, and 42.5% (*n* = 607) reported they had enough finances, but no extras. Most of the sample reported identifying as heterosexual (*n* = 1099, 77%), and the sexual minority category with the highest proportion of participants was bisexual (*n* = 158, 11.1%).

More than a third of participants rated their mental health (*n* = 506), and emotional health as “fair” (*n* = 478). Nearly half of the sample reported fairly bad or very bad sleep quality during the past month (*n* = 686). In terms of religiosity, more than half of the sample reported being very or somewhat religious (*n* = 872). The self-rated skin tone with the highest proportion of participants was dark (*n* = 471, 33%), and the lowest was very light (*n* = 15, 1.1%). Half of the participants reported poor SRH (*n* = 716), and 25% reported worse RSRH compared to most people their age.

More than half of the participants reported having someone in their life who provides them with emotional support (*n* = 959) and social network support (*n* = 805). Approximately a quarter of participants reported participating in volunteer activities during the past year (*n* = 346), and 29% reported providing financial support to people who did not live with them (*n* = 407). Nearly 40% of participants reported providing non-financial support to others who did not live with them (*n* = 547). Most participants reported never being incarcerated (*n* = 1155) and of those with a current partner (*n* = 902), nearly 75% indicated that their partner had never been incarcerated (*n* = 664). A majority of participants reported low overall levels of trust (*n* = 1093). More than 60% of the participants reported experiencing high (between 4 and 16) adverse childhood experiences (ACES) (*n* = 875), with only 32% reporting no or low ACES (between 0 and 3).

More than 30% of participants reported experiencing gentrification in their current neighborhood (*n* = 449), and of those, 16% perceived benefits of it (*n* = 72), and 32% reported being negatively impacted by it (*n* = 143). SECURE study enrollment was highest in the year 2022 (*n* = 535, 37.5%), and the lowest in 2021 (*n* = 145; 10.2%). More than 75% of the participants reported currently living in Wayne County (*n* = 1088), with 9.3% residing in Macomb (*n* = 133) and 11.5% in Oakland (*n* = 164) County. Sixty percent of participants were currently renting their home (*n* = 855) and nearly 60% were currently employed (*n* = 821).

### Descriptive Statistics for Eviction and Housing Discrimination Experiences

A quarter of the study sample reported experiencing an eviction before their 18th birthday (*n* = 354). Over 40% of the sample reported ever experiencing housing discrimination (*n* = 588). More than half of the study sample reported ever experiencing a court-ordered (*n* = 432) and/or an illegal eviction (*n* = 360). Of the evictions ever experienced, 54.5% were court-ordered (*n* = 432) and 45.5% were illegal (*n* = 360). In the past 2 years, 15.2% of the sample reported experiencing an eviction. Of the evictions experienced in the past 2 years, 56.2% were court-ordered (*n* = 122) and 43.8% were illegal (*n* = 95). All correlations between the five eviction variables were different from 0 (*p* < 0.02 for all, data not shown). The strongest correlation was between ever experiencing an eviction and experiencing a court ordered eviction in the past 2 years (*r* = 0.35, *p* < 0.0001). The weakest correlation was between eviction during childhood and court-ordered eviction in the past 2 years (*r* = 0.06; *p* = 0.02).

### Descriptive Statistics Stratified by Five Eviction Experiences

In bivariate analyses, age, RSRH, and financially supporting others was associated with experiencing an eviction before age 18. Relationship status and self-reported skin tone was associated with ever reporting court-ordered eviction. Educational attainment, self-rated sleep quality, and overall trust level was associated with childhood, ever and past 2 years court-ordered, and ever illegal eviction. All five eviction measures were associated with annual income, financial status today, self-rated mental and emotional health, social network support, housing tenure, and ever experiencing housing discrimination. Sexual orientation and enrollment year was associated with illegal eviction in the past 2 years. The only descriptive variable that was associated with both childhood eviction and ever experiencing court-ordered eviction was SRH. Having emotional support, and ever being incarcerated was associated with all four adulthood eviction measures. Volunteering in the past year and employment status was associated with court-ordered eviction in the past 2 years. Providing non-financial support to others was associated with childhood eviction, and ever experiencing both court ordered and illegal eviction. The only descriptive variable that was associated with both ever experiencing a court-ordered eviction and ever experiencing an illegal eviction was current partner ever being incarcerated. Current neighborhood gentrification and number of ACES were both associated with all eviction measures except court-ordered eviction in the past 2 years. The only descriptive variable that was associated with ever and past 2 years court-ordered eviction was perceived disadvantages of neighborhood gentrification. We observed no strong evidence of bivariate associations between any of the five eviction measures we examined and the following variables: religiosity, perceived neighborhood gentrification benefit, number of live born children, and current county of residence (Table 2).

**Table 2:** Descriptive statistics, stratified by five eviction experiences: Social Epidemiology to combat Unjust Residential Evictions (SECURE) study (*n* = 1428; 2021–2024)

Characteristic	Total sample (*N* = 1428; 100%) *N* (%)	Eviction before age 18 (*n* = 354; 24.8%) *N* (%)	*p*	Ever court ordered eviction (*n* = 432; 30.3%) *N* (%)	*p*	Court ordered eviction in past 2 years (*n* = 122; 8.54%) *N* (%)	*p*	Ever illegal eviction (*n* = 360; 25.2%) *N* (%)	*p*	Illegal eviction in past 2 years (*n* = 95; 6.7%) *N* (%)	*p*
Age			< .0001		0.0606		0.1982		0.1278		0.2049
18–19	12 (0.8)	3 (0.9)		1 (0.2)		1 (0.8)		1 (0.3)		1 (1.1)	
20–24	87 (6.1)	32 (9)		17 (3.9)		10 (8.2)		13 (3.6)		8 (8.4)	
25–29	220 (15.4)	74 (21)		62 (14.4)		26 (21.3)		59 (16.4)		19 (20)	
30–34	355 (24.9)	95 (26.8)		107 (24.8)		31 (25.4)		87 (24.2)		28 (29.5)	
35 +	754 (52.8)	150 (42.4)		245 (56.7)		54 (44.3)		200 (56.6)		39 (41.1)	
Missing	0 (0)										
Relationship status			0.0470		0.0045		0.0651		0.1821		0.0537
Married	273 (19.1)	71 (20.1)		58 (13.4)		12 (9.8)		62 (17.2)		9 (9.5)	
Living with partner	231 (16.2)	53 (15)		67 (15.5)		20 (16.4)		50 (13.9)		16 (16.8)	
Divorced	133 (9.3)	19 (5.4)		40 (9.3)		11 (9)		34 (9.4)		8 (8.4)	
Separated	59 (4.1)	16 (4.5)		18 (4.2)		8 (6.6)		21 (5.8)		8 (8.4)	
Widowed	12 (0.8)	2 (0.6)		5 (1.2)		2 (1.6)		2 (0.6)		1 (1.1)	
Never married	715 (50.1)	192 (54.4)		244 (56.5)		69 (56.6)		191 (53.1)		53 (55.8)	
Missing	5 (0.4)										
Educational attainment			0.0001		< .0001		0.0452		< .0001		0.1698
1–8th grade	1 (0.1)	1 (0.3)		1 (0.2)		0 (0)		1 (0.3)		0 (0)	
9–11th grade	99 (6.9)	35 (9.9)		30 (6.9)		11 (9)		38 (10.6)		10 (10.5)	
High school diploma	216 (15.1)	65 (18.4)		77 (17.8)		27 (22.1)		55 (15.3)		19 (20)	
GED	93 (6.5)	32 (9)		33 (7.6)		8 (6.6)		30 (8.3)		8 (8.4)	
Some college	495 (34.7)	116 (32.8)		168 (38.9)		46 (37.7)		136 (37.8)		35 (36.8)	
Associate’s degree	162 (11.3)	42 (11.9)		55 (12.7)		11 (9)		53 (14.7)		7 (7.4)	
Bachelor’s degree	203 (14.2)	30 (8.5)		41 (9.5)		16 (13.1)		27 (7.5)		13 (13.7)	
Graduate degree	138 (9.7)	32 (9)		26 (6)		2 (1.6)		18 (5)		3 (3.2)	
Doctorate	17 (1.2)	1 (0.3)		1 (0.2)		1 (0.8)		2 (0.6)		0 (0)	
Missing	4 (0.3)										
Total Income last year			0.0004		0.0004		< .0001		0.0007		0.0033
< $35,000	793 (55.5)	227 (64.1)		273 (63.2)		93 (76.2)		229 (63.6)		67 (70.5)	
≥ $35,000	619 (43.4)	127 (35.9)		159 (36.8)		29 (23.8)		131 (36.4)		28 (29.5)	
Missing	16 (1.1)										
Financial situation today			0.0001		< .0001		< .0001		< .0001		0.0004
Very poor, not enough to get by	160 (11.2)	49 (13.8)		58 (13.4)		25 (20.5)		60 (16.7)		18 (19)	
Barely enough to get by	417 (29.2)	131 (37)		154 (35.7)		54 (44.3)		129 (35.8)		36 (37.9)	
Have enough, but no extras	607 (42.5)	133 (37.6)		178 (41.2)		40 (32.8)		141 (39.2)		39 (41.1)	
Have more than enough	193 (13.5)	36 (10.2)		34 (7.9)		2 (1.6)		24 (6.7)		2 (2.1)	
Well to do	40 (2.8)	5 (1.4)		8 (1.9)		1 (0.8)		6 (1.7)		0 (0)	
Missing	11 (0.8)										
Sexual orientation			0.4781		0.1480		0.2045		0.0513		0.0426
Asexual	69 (4.8)	21 (6)		24 (5.6)		9 (7.4)		25 (7)		5 (5.3)	
Bisexual	158 (11.1)	41 (11.7)		53 (12.4)		16 (13.2)		46 (12.9)		13 (13.8)	
Gay or lesbian	34 (2.4)	7 (2)		11 (2.6)		6 (5)		10 (2.8)		3 (3.2)	
Heterosexual	1099 (77.0)	265 (75.5)		320 (74.8)		86 (71.1)		261 (72.9)		65 (69.2)	
Pansexual or demisexual	29 (2.0)	10 (2.9)		14 (3.3)		3 (2.5)		11 (3.1)		5 (5.3)	
Queer	9 (0.6)	2 (0.6)		4 (0.9)		1 (0.8)		3 (0.8)		0 (0)	
Questioning or unsure	14 (1.0)	5 (1.4)		2 (0.5)		0 (0)		2 (0.6)		3 (3.2)	
Missing	16 (1.1)										
Self-rated mental health			< .0001		0.0009		0.0011		0.0004		0.0008
Excellent	107 (7.5)	23 (6.5)		29 (6.7)		7 (5.7)		25 (7)		6 (6.3)	
Very good	177 (12.4)	34 (9.6)		38 (8.8)		11 (9)		30 (8.4)		4 (4.2)	
Good	408 (28.6)	82 (23.2)		118 (27.4)		28 (23)		94 (26.3)		25 (26.3)	
Fair	506 (35.4)	141 (39.9)		171 (39.7)		45 (36.9)		142 (39.7)		35 (36.8)	
Poor	186 (13.0)	73 (20.7)		75 (17.4)		31 (25.4)		67 (18.7)		25 (26.3)	
Missing	44 (3.1)										
Self-rated emotional health			< .0001		0.0019		0.0007		0.0012		0.0006
Excellent	101 (7.1)	18 (5.1)		23 (5.3)		5 (4.1)		23 (6.4)		5 (5.3)	
Very good	203 (14.2)	33 (9.4)		52 (12.1)		11 (9)		36 (10.1)		5 (5.3)	
Good	409 (28.6)	99 (28.1)		113 (26.2)		29 (23.8)		93 (26)		20 (21.1)	
Fair	478 (33.5)	135 (38.2)		170 (39.4)		46 (37.7)		143 (39.9)		42 (44.2)	
Poor	193 (13.5)	68 (19.3)		73 (16.9)		31 (25.4)		63 (17.6)		23 (24.2)	
Missing	44 (3.1)										
Self-rated sleep quality			0.0137		0.0007		0.0259		< .0001		0.0750
Very good	105 (7.4)	24 (6.9)		25 (6.1)		6 (5)		18 (5.2)		3 (3.5)	
Fairly good	551 (38.6)	120 (34.7)		143 (34.6)		37 (31.1)		116 (33.6)		31 (35.6)	
Fairly bad	487 (34.1)	138 (39.9)		170 (41.2)		51 (42.9)		143 (41.5)		42 (48.3)	
Very bad	199 (13.9)	64 (18.5)		75 (18.2)		25 (21)		68 (19.7)		11 (12.6)	
Missing	86 (6.0)										
Religiosity			0.3890		0.8013		0.0744		0.1076		0.9214
Very religious	224 (15.7)	51 (14.4)		68 (15.8)		27 (22.1)		64 (17.8)		14 (14.7)	
Somewhat religious	648 (45.4)	159 (44.9)		201 (46.6)		51 (41.8)		173 (48.1)		43 (45.3)	
Not too religious	331 (23.2)	83 (23.5)		95 (22)		22 (18)		69 (19.2)		22 (23.2)	
Not at all religious	212 (14.9)	61 (17.2)		67 (15.6)		22 (18)		54 (15)		16 (16.8)	
Missing	13 (0.9)										
Self-reported skin tone			0.1533		0.0343		0.2762		0.8228		0.5616
Very light	15 (1.1)	3 (0.9)		4 (0.9)		2 (1.7)		3 (0.8)		0 (0)	
Light	30 (2.1)	7 (2)		8 (1.9)		0 (0)		6 (1.7)		2 (2.1)	
Beige	114 (8.0)	29 (8.2)		36 (8.3)		11 (9.1)		27 (7.5)		9 (9.5)	
Medium	423 (29.6)	109 (30.8)		117 (27.1)		36 (29.8)		106 (29.5)		32 (33.7)	
Tan	314 (22.0)	60 (17)		84 (19.4)		20 (16.5)		75 (20.9)		22 (23.2)	
Dark	471 (33.0)	135 (38.1)		173 (40.1)		49 (40.5)		128 (35.7)		25 (26.3)	
Very deep	43 (3.0)	11 (3.1)		10 (2.3)		3 (2.5)		14 (3.9)		5 (5.3)	
Missing	18 (1.3)										
Self-rated health			0.0421		0.0260		0.5000		0.3235		0.2176
Good	625 (43.8)	145 (41.9)		174 (42.1)		52 (43.7)		153 (44.4)		35 (40.2)	
Poor	716 (50.1)	201 (58.1)		239 (57.9)		67 (56.3)		192 (55.7)		52 (59.8)	
Missing	87 (6.1)										
Relative self-rated health			0.0033		0.3664		0.0933		0.0842		0.9243
Better/average	986 (69.1)	233 (67.7)		296 (72)		80 (67.2)		241 (70.1)		63 (73.3)	
Worse	352 (24.7)	111 (32.3)		115 (28)		39 (32.8)		103 (29.9)		23 (26.7)	
Missing	90 (6.3)										
Emotional support			0.1046		0.0007		0.0149		0.0004		< .0001
No	392 (27.5)	113 (32.4)		147 (35.3)		46 (38.7)		127 (36.4)		43 (48.3)	
Yes	959 (67.2)	236 (67.6)		269 (64.7)		73 (61.3)		222 (63.6)		46 (51.7)	
Missing	77 (5.4)										
Social network support			< .0001		< .0001		< .0001		< .0001		0.0238
No	544 (38.1)	180 (51.6)		212 (51)		73 (61.3)		176 (50.4)		46 (51.7)	
Yes	805 (56.4)	169 (48.4)		204 (49)		46 (38.7)		173 (49.6)		43 (49.3)	
Missing	79 (5.5)										
Volunteer past year			0.5050		0.6813		0.0113		0.4472		0.9137
No	1004 (70.3)	255 (73.1)		311 (75.1)		100 (84)		263 (75.8)		65 (73.9)	
Yes	346 (24.2)	94 (26.9)		103 (24.9)		19 (16)		84 (24.2)		23 (26.1)	
Missing	78 (5.5)										
Financially support others			0.0016		0.0545		0.4262		0.1576		0.9365
No	1008 (70.6)	228 (64.6)		293 (67.8)		83 (68)		246 (68.3)		68 (71.6)	
Yes	407 (28.5)	125 (35.4)		139 (32.2)		39 (32)		114 (31.7)		27 (28.4)	
Missing	13 (0.9)										
Non-financially support others			0.0291		0.0114		0.9762		0.0014		0.2845
No	863 (60.4)	197 (56.1)		241 (56.1)		74 (61.2)		194 (54)		53 (55.8)	
Yes	547 (38.3)	154 (43.9)		189 (44)		47 (38.8)		165 (46)		42 (44.2)	
Missing	18 (1.3)										
Self ever incarcerated			0.3187		< .0001		0.0006		< .0001		0.0005
No	1155 (80.9)	293 (84.9)		327 (79.6)		90 (76.3)		269 (78.9)		63 (74.1)	
Yes	181 (12.7)	52 (15.1)		84 (20.4)		28 (23.7)		72 (21.1)		22 (25.9)	
Missing	92 (6.4)										
Partner ever incarcerated**			0.1175		< .0001		0.1153		0.0011		0.5047
No	664 (73.6)	164 (68.3)		162 (60.7)		49 (64.5)		150 (64.1)		39 (68.4)	
Yes	256 (28.4)	76 (31.7)		105 (39.3)		27 (35.5)		84 (35.9)		18 (31.6)	
Missing	94 (6.6)										
Overall trust			0.0224		0.0316		0.0211		0.0275		0.4627
Low (you can’t be too careful)	1093 (76.5)	296 (85.3)		350 (84.5)		106 (89.1)		296 (85.1)		74 (84.1)	
High (people can be trusted)	254 (17.8)	51 (14.7)		64 (15.5)		13 (10.9)		52 (14.9)		14 (15.9)	
Missing	81 (5.7)										
Current neighborhood gentrification			0.0169		0.0380		0.3352		0.0002		0.0024
No	949 (66.5)	222 (62.7)		275 (63.8)		78 (63.9)		215 (59.7)		51 (53.7)	
Yes*	449 (31.4)	132 (37.3)		156 (36.2)		44 (36.1)		145 (40.3)		44 (46.3)	
Missing	30 (2.1)										
Perceived gentrification disadvantage*			0.3909		0.0248		0.0087		0.2126		0.7171
No	303 (67.5)	84 (64.6)		93 (60.8)		22 (50)		91 (63.6)		28 (65.1)	
Yes	143 (31.8)	46 (35.4)		60 (39.2)		22 (50)		52 (36.4)		15 (34.9)	
Missing	7 (0.5)										
Perceived gentrification benefit*			0.0889		0.8603		0.0739		0.2427		0.6671
No	376 (83.7)	116 (88.6)		128 (84.2)		41 (93.2)		124 (86.7)		37 (86.1)	
Yes	72 (16.0)	15 (11.5)		24 (15.8)		3 (6.8)		19 (13.3)		6 (14)	
Missing	6 (0.4)										
Number of live born children			0.2449		0.0580		0.0721		0.0981		0.2767
0	131 (9.2)	28 (7.9)		31 (7.2)		6 (4.9)		26 (7.3)		6 (6.3)	
1 +	1261 (88.3)	326 (92.1)		397 (92.8)		116 (95.1)		331 (92.7)		89 (93.7)	
Missing	36 (2.5)										
Number of adverse childhood experiences			< .0001		0.0003		0.2418		< .0001		< .0001
0–3	461 (32.3)	60 (17.4)		109 (26.5)		33 (28)		75 (21.9)		20 (23.3)	
4–7	373 (26.1)	87 (25.2)		121 (29.4)		34 (28.8)		81 (23.7)		19 (22.1)	
8–11	313 (21.9)	102 (29.6)		114 (27.7)		28 (23.7)		95 (27.8)		19 (22.1)	
12–16	189 (13.2)	96 (27.8)		68 (16.5)		23 (19.5)		91 (26.6)		28 (32.6)	
Missing	92 (6.4)										
Current county of residence			0.4624		0.4243		0.7131		0.1300		0.3338
Wayne	1088 (76.2)	280 (79.6)		345 (80.4)		95 (77.9)		287 (80)		78 (83)	
Macomb	133 (9.3)	28 (8)		40 (9.3)		14 (11.5)		25 (7)		5 (5.3)	
Oakland	164 (11.5)	44 (12.5)		44 (10.3)		13 (10.7)		47 (13.1)		11 (11.7)	
Enrollment year			0.3527		0.4278		0.3315		0.0511		0.0184
2021	145 (10.2)	35 (9.9)		42 (9.7)		7 (5.7)		27 (7.5)		9 (9.5)	
2022	535 (37.5)	127 (35.9)		176 (40.7)		46 (37.7)		131 (36.4)		24 (25.3)	
2023	442 (31.0)	123 (34.8)		129 (29.9)		38 (31.2)		131 (36.4)		42 (44.2)	
2024	306 (21.4)	69 (19.5)		85 (19.7)		31 (25.4)		71 (19.7)		20 (21.1)	
Missing	0 (0)										
Current housing tenure			0.0110		< .0001		< .0001		0.0015		0.0140
Occupied without payment of rent	50 (3.5)	13 (3.7)		15 (3.5)		5 (4.1)		14 (3.9)		4 (4.2)	
Other	66 (4.6)	21 (5.9)		26 (6)		13 (10.7)		20 (5.6)		10 (10.5)	
Owned free and clear	172 (12)	40 (11.3)		48 (11.1)		7 (5.7)		40 (11.1)		9 (9.5)	
Owned with a mortgage or loan	266 (18.6)	46 (13)		46 (10.7)		4 (3.3)		43 (11.9)		10 (10.5)	
Rented	855 (59.9)	234 (66.1)		297 (68.8)		93 (76.2)		243 (67.5)		62 (65.3)	
Missing	19 (1.33)										
Employment status			0.3295		0.2709		0.0005		0.2179		0.0536
Unemployed	591 (41.4)	157 (44.4)		191 (44.3)		69 (57)		161 (45)		49 (51.6)	
Employed	821 (57.5)	197 (55.7)		240 (55.7)		52 (43)		197 (55)		46 (48.4)	
Missing	16 (1.1)										
Housing discrimination ever			< .0001		< .0001		0.0010		< .0001		< .0001
No	819 (57.4)	156 (44.2)		193 (44.8)		54 (44.3)		146 (40.7)		36 (38.3)	
Yes	588 (41.2)	197 (55.8)		238 (55.2)		68 (55.7)		213 (59.3)		58 (61.7)	
Missing	21 (1.5)										

Missing data for eviction during childhood, ever court-ordered eviction, court-ordered eviction in the past 2 years, and illegal eviction in the past 2 years: (*n* = 44, 3.1%); missing data for ever illegal eviction: (*n* = 47, 3.29%)

^**^Among those who reported currently having a partner (*n* = 920)

*p*-values represent chi square test of difference across levels of descriptive variables by each measure of eviction

### Associations Between Eviction Experiences and SRH and RSRH

We observed higher risk of poor SRH (RR 1.12; CI 1.01, 1.25) and worse RSRH (RR1.34; 95% CI 1.11, 1.62) for women who reported experiencing an eviction before their 18th birthday (versus those who did not), in fully adjusted models (Table 3). Similarly, ever (compared to never) experiencing an illegal eviction was associated with higher risk of poor SRH (RR 1.17; 95% CI 1.02, 1.34) and worse RSRH (RR 1.37; 95% CI 1.07, 1.76), in adjusted models. Ever experiencing a court-ordered eviction (versus never) was associated with higher risk of poor SRH (RR 1.16; 95% CI 1.01, 1.32) in adjusted models. No strong evidence of association was observed for court-ordered or illegal eviction experiences in the past 2 years and risk of poor SRH or worse RSRH in unadjusted or adjusted models.

**Table 3:** Associations between five eviction measures and risk of poor SRH and worse RSRH, Social Epidemiology to Combat Unjust Residential Evictions (SECURE) study, 2021–2024

	Self-rated health		Relative self-rated health	
Exposure	Unadjusted RR (95% CI)	Adjusted RR (95% CI)	Unadjusted RR (95% CI)	Adjusted RR (95% CI)
Eviction before 18 years old	1.12 (1.01, 1.25)	1.12 (1.01, 1.25)	1.34 (1.11, 1.61)	1.34 (1.11, 1.62)
Court ordered eviction—ever	1.16 (1.02, 1.32)	1.16 (1.01, 1.32)	1.10 (0.87, 1.41)	1.11 (0.87, 1.43)
Court ordered eviction—past 2 years	1.01 (0.80, 1.26)	1.02 (0.82, 1.28)	1.20 (0.83, 1.73)	1.19 (0.82, 1.75)
Illegal eviction—ever	1.15 (1.01, 1.32)	1.17 (1.02, 1.34)	1.37 (1.08, 1.75)	1.37 (1.07, 1.76)
Illegal eviction—past 2 years	1.13 (0.88, 1.44)	1.13 (0.88, 1.44)	1.28 (0.83, 1.98)	1.30 (0.84, 2.00)

*RR* relative risk, *CI* confidence interval

^*^Missing data for self-rated health *n* = 87 (6.1%), and relative self-rated health *n* = 90 (6.3%)

Models adjusted for age, income, employment status, housing tenure, number of live born children; sample size for models of eviction before 18 years old and self-rated health and relative self-rated health (*n* = 1074), sample size for models of ever and past 2 years court-ordered and illegal eviction, and self-rated health and relative self-rated health (*n* = 1428)

## Discussion

The main findings of this study were that eviction during childhood, and adulthood court-ordered and illegal eviction were highly prevalent among SECURE study participants, and these experiences were associated with poor SRH and worse RSRH. Specifically, 50% of participants reported ever experiencing a court-ordered and/or or illegal eviction, and 25% experienced an eviction during childhood. Nearly half of the evictions ever experienced by our study population were illegal. Further, we report that childhood, and ever experiencing court-ordered, and ever experiencing illegal evictions were associated with between 12% and 17% higher risk of poor SRH. We also report that childhood eviction and ever experiencing an illegal eviction were associated with between 34% and 37% higher risk of worse RSRH.

The role that discrimination plays in the eviction crisis is understudied [25, 26], and calls have been made for more research that focuses explicitly on quantifying individual experiences of discrimination in housing and its impact on health [26]. Unfortunately, more than 40% of SECURE study participants reported ever experiencing housing discrimination. In one of few existing studies, using data from Philadelphia from 2008, while only 5% of respondents reported experiencing housing discrimination, the descriptive statistics reported suggests possible links with a range of socio-demographic and social factors, as well as adverse health outcomes [27]. Future studies should assess the impact of housing discrimination on the mental and physical health of Black reproductive age women.

Global estimates of the prevalence of four or more ACEs is 13% [28]. Surprisingly, more than 60% of SECURE study participants reported experiencing between 4 and 16 ACEs. This underscores how important study populations like ours are to understanding risk and protective factors for population health outcomes among groups made vulnerable by structural racism across the life-course. Notably, experiencing an eviction before age 18 was not included in the original ACE nor the expanded ACE scales, but a quarter of SECURE study participants reported having this experience. We suggest that the expanded ACEs scale be updated to include residential eviction during childhood. Future research and advocacy should focus on reducing risk of ACEs among Black children, including mitigating harms and addressing root causes of housing instability caused by residential eviction.

The small existing literature on associations between eviction and SRH reports mixed findings [2, 21, 29–31]. The existing studies include samples with low prevalence of eviction, do not include multiple types of eviction, are challenged by limited available data that is often dated, and are not focused explicitly on the group made most vulnerable to residential eviction by structural racism and sexism. Our work adds critical new evidence on the experience of eviction, by life stage (childhood and adulthood), timing (ever and past two years), and type (court-ordered and illegal) among Black reproductive age women. In the current study, we also present distributions of other important socio-demographic factors and health outcomes in our newly formed and well-characterized study population, which includes a large proportion of people who experienced housing instability and are traditionally left out of large-scale research projects. Future mixed-methods community-based participatory action research on the complex nature of court-ordered and illegal eviction will facilitate naming who and what is responsible [8] for inequities in Black maternal health [32], to support effective interventions including policy solutions.

### Limitations

SECURE study has a cross-sectional design, and we cannot make causal estimates or rule out the possibility of recall bias. However, social desirability bias may have been mitigated because the online survey was self-administered for most participants, and the remaining participants completed the survey via telephone rather than in-person. To be sure, there is no evidence that Black women are more likely than people with different identities to mis-recall life events in survey-based research studies. Given our purposive community-based sampling strategy, we cannot rule out selection bias, especially if poor health status precluded participation, or if prior health conditions occurred before eviction experiences. Future research with a longitudinal design could account for poor health status before eviction, as well as the health status of the parents or caregivers of children before they experienced eviction, as well as other important time varying individual and community level covariates. Given that 50% of our participants reported experiencing an eviction during their lifetime, it is unlikely that our research suffered selection bias based on history of eviction. The SECURE study used convenience sampling, which may limit generalizability of our results to populations with different sociodemographic, social, and geographic characteristics. However, our study sample had similar sociodemographic characteristics as Black women in Wayne County, MI, the State of Michigan, as well as the USA according to 1-year population profile data from 2021 from the American Community Survey (data available upon request) which supports the representativeness of our study sample to the target population, and provides new and relevant information to inform public health, clinical care, and policymaking to support population health equity and social justice.

### Strengths

SECURE study is, to our knowledge, the first and most comprehensive data source on the magnitude and severity of court-ordered and illegal eviction as well as the impacts of residential eviction experiences on the health and wellbeing of reproductive age Black women, families, and communities. This study provides new insights into the burden of illegal evictions, which are highly prevalent but understudied given existing data limitations [33]. Our high survey response rate and minimal missing data supports the internal validity of our data. Our extensive verification process minimized threats to external validity, in that only confirmed Black women participated. Our data collection process and our experience with fraudulent participation attempts raises questions about the validity of online survey recruitment efforts that do not involve a human verification of eligibility step. Our study design, which used community based participatory action [18] research principles, allowed us to successfully encourage high study participation among those who have experienced an eviction. As a result, our data overcomes the limitations of existing studies in which people who have experienced housing instability are less likely to participate in research. Our results highlight the durability of the negative health impacts of eviction during childhood, as well as connections between ever experiencing court-ordered and illegal evictions and adult SRH and RSRH among Black reproductive age women.

## Conclusion

Residential eviction among Black reproductive age women is a critical public health issue. Future advocacy, interventions, and policy solutions are warranted. Given the broad range and depth of the SECURE study data collection, and that *n* = 27 of the *n* = 31 descriptive variables presented here were associated with one or all five of our eviction measures, we plan to systematically and rigorously examine novel research questions in the order determined by our community advisory board, participants, and relevant interested parties. We urge funders to support more novel community-partnered research using participatory action research methods [18] to advance health equity goals nationally and internationally, and ensure the human right to housing for all.

## Data Availability

SECURE Study Data is not publicly available, and may be available by request to the principal investigator (Dr. SSJ).
